# Solvent Performance Evaluation of Heavy Oil in Coal–Oil Co-Liquefaction

**DOI:** 10.3390/ijms26136048

**Published:** 2025-06-24

**Authors:** Guanghua Yang, Juan Ma, Caitao Chen, Tingting Cui, Yingluo He, Ting Liu

**Affiliations:** 1 State Key Laboratory of Chemistry and Utilization of Carbon Based Energy Resources, College of Chemistry, Xinjiang University, Urumqi 830017, China; ygh5436438@163.com (G.Y.); kjcmj@xju.edu.cn (J.M.); chenct80854@163.com (C.C.); tingtingcui2001@163.com (T.C.); 2 Department of Applied Chemistry, School of Engineering, University of Toyama, Gofuku 3190, Toyama 930-8555, Japan

**Keywords:** coal–oil co-liquefaction, heavy oil, column chromatography, polycyclic aromatic hydrocarbons

## Abstract

In this study, we investigated the solvent performance of six heavy oils from Xinjiang, China, for coal–oil co-liquefaction (COCL). Autoclave experiments revealed that shale oil vacuum residue (SOVR) provided the best liquefaction performance. The oils were characterized using FT-IR, ^13^C-NMR, ^1^H-NMR, and column chromatography, which revealed that they were mainly composed of aliphatic compounds, with minor aromatic and substituted aromatic compounds. The pyrolytic degradation quality indices (PDQIs), solubility parameter (δ_C_), and polycyclic aromatic hydrocarbon content (H_A2_ + H_A3_) were calculated and correlated with liquefaction performance. The results showed a strong linear relationship between H_A2_ + H_A3_ and oil yield (R^2^ = 0.90), and the aromatic content (AR) was also positively related to oil yield. This study suggests that AR content and H_A2_ + H_A3_ are effective indicators for evaluating the solvent performance of heavy oils in COCL.

## 1. Introduction

Direct coal liquefaction (DCL) is a process through which high-value liquid fuel is produced by combining free radical fragments generated during coal pyrolysis with hydrogen radicals [1,2,3]. Coal–oil co-liquefaction (COCL) is based on DCL, and heavy oil is used as a solvent to promote the conversion of coal into light oil through hydrogenation [4]. This process simultaneously transforms coal and heavy oil, improves the quality of oil products, and reduces carbon emissions [5]. As a solvent, heavy oil not only promotes coal pyrolysis but also possesses hydrogen transfer capabilities. Therefore, the solvent properties of heavy oil play a crucial role in COCL [6]. However, there are relatively few methods for evaluating the solvent performance of heavy oil in COCL. Therefore, it is of great significance to explore the methods used to evaluate the solvent performance of heavy oil for the reasonable matching of coal and heavy oil in the COCL process.

Previous reports have examined evaluation methods for recycle solvent. Painter et al. [7] evaluated the liquefaction ability of recycle solvent in terms of its dissolution capacity for coal. They calculated the dissolution parameters (δ_C_) for both coal and recycle solvents, indicating that the closer the δ_C_ values, the better the dissolution effect and the more complete the liquefaction reaction. This method is based on the decomposition of coal in the recycle solvent, which is a physical interaction [8,9]. However, coal and heavy oil undergo chemical reactions in the COCL process. Rahimi et al. [10] utilized ^13^H-NMR to analyze recycle solvent, confirming that hydrogen atoms within the chemical shift range of 1.9 to 2.9 ppm in ^1^H-NMR are available hydrogen, which is related to liquefaction performance. Shen et al. [11] used the ^1^H-NMR method to compute pyrolytic degradation quality indices (PDQIs) in order to evaluate the hydrogen supply capacity of recycle solvent, and they found a positive correlation between the PDQIs value of recycle solvents and the yield of coal liquefaction oil. Niu et al. [12] calculated the dissociation energies of C-H in hydrogenated aromatics and H-H in H_2_ within recycle solvent, and they discovered that hydrogenated aromatics are more readily able to supply hydrogen to coal pyrolysis free radicals than H_2_. The main compounds in recycle solvent are aromatic hydrocarbons, which mainly exist in the form of hydrogenated aromatic hydrocarbons [13]. However, heavy oil contains many structures other than hydrogenated aromatic hydrocarbons. Therefore, this method may not be suitable as an evaluation method for the solvent properties of heavy oil in COCL. Flynn et al. [14] used five types of residual oil as liquefaction solvents, and they found that residual oil with a high aromatic content exhibited a higher liquefaction rate when used as a coal liquefaction solvent. Given the differences in the compositional structure and mechanism of action within coal liquefaction systems between recycle solvent and heavy oil, current evaluation methods for recycle solvents may not be suitable for heavy oil. Additionally, there are relatively few studies on the methods used to evaluate the solvent performance of heavy oil in COCL. Therefore, it is necessary to explore the evaluation methods suitable for heavy oil.

In this study, six typical heavy oils from Xinjiang, namely shale oil vacuum residue (SOVR), multi-component hydrocarbon (MCH), shale oil (SO), heavy oil of northern Xinjiang (HONX), heavy oil of southern Xinjiang (HOSX), and oil sands oil (OSO), were selected for combination with Naomaohu coal (NMHC) in COCL experiments to explore their solvent performances. First, the structural characteristics of the six heavy oils were analyzed using a component analysis, FT-IR, ^13^C-NMR, and ^1^H-NMR. Then, the six heavy oils were used as solvents in COCL experiments to investigate their solvent properties. The pyrolytic degradation quality indices (PDQIs), solubility parameter (δ_C_), AR content, and polycyclic aromatic hydrocarbon content (H_A2_ + H_A3_) of the six heavy oils were correlated with the oil yield of COCL to evaluate their performance as solvents. The aim of this study was to develop an appropriate evaluation method for the solvent performance of heavy oils for COCL, providing scientific guidance for the reasonable matching of coal types and heavy oils, as well as improving the efficiency of COCL.

## 2. Results

### 2.1. Column Chromatography and Co-Liquefaction Experiments of Heavy Oil

#### 2.1.1. Analysis of Group Component Contents in Heavy Oil

**Figure 1:**
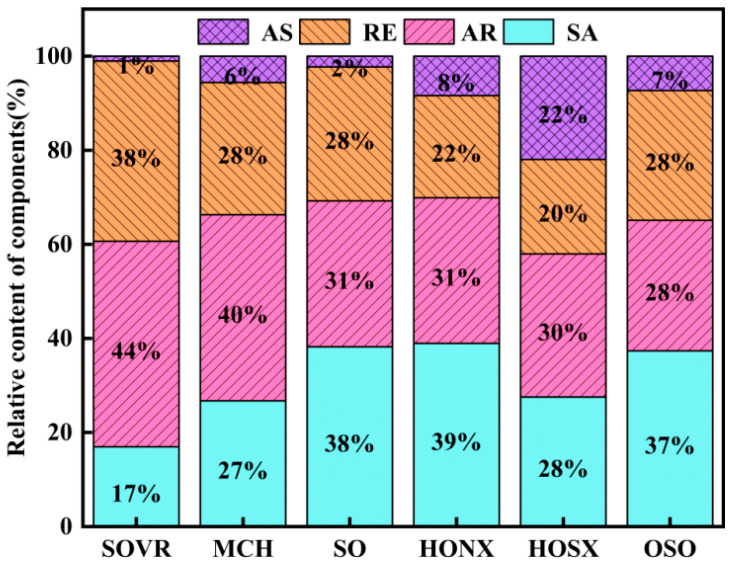
Group component contents in heavy oil.

The saturation (SA), aromatic (AR), resin (RE), and asphaltene (AS) contents in the six heavy oils were separated using column chromatography, and the separation process is shown in Figure S1. The group component contents in the heavy oils are shown in Figure 1. It was found that the AR content in the SOVR and MCH was much higher than that in the other four oil samples; in particular, the AR content in SOVR was highest, while that in the other four heavy oils was similar. The SA content in SO, HONX, and OSO was relatively high, among which it was highest in HONX. The SA content in MCH and HOSX was similar, and it was lowest in SOVR. The RE content in SOVR was highest, while that in MCH, SO, and OSO was similar. The AS content in MCH, HONX, and OSO was similar, while that in SOVR and SO was low. Among the six heavy oils, the AS content in HOSX was highest.

The group composition of heavy oil has different effects on liquefaction performance. AR in heavy oil contains polycyclic and hydrogenated aromatic hydrocarbons, has hydrogen transfer and supply abilities [15], and is beneficial for the liquefaction reaction. Although SA can improve the fluidity of the reaction system [16], it cannot provide active hydrogen. On the contrary, SA cracks in COCL, thus competing for active hydrogen in the system [17], inhibiting AS cracking, and leading to coking. RE stabilizes the dispersion of the system during the liquefaction reaction, prevents coal from settling, and contributes to the hydrogen transfer of aromatic components [18]. AS has a large molecular weight, is not easily converted, and is easy to coke in the reaction, which is not conducive to liquefaction [7]. As shown in Figure 1, SOVR is characterized by high AR and RE contents and a low SA content. Therefore, it can be inferred that SOVR will have a higher oil yield in COCL. Conversely, OSO contains a higher SA content and lower AR and RE contents; therefore, it may have a lower oil yield in COCL.

#### 2.1.2. Co-Liquefaction Performance

**Figure 2:**
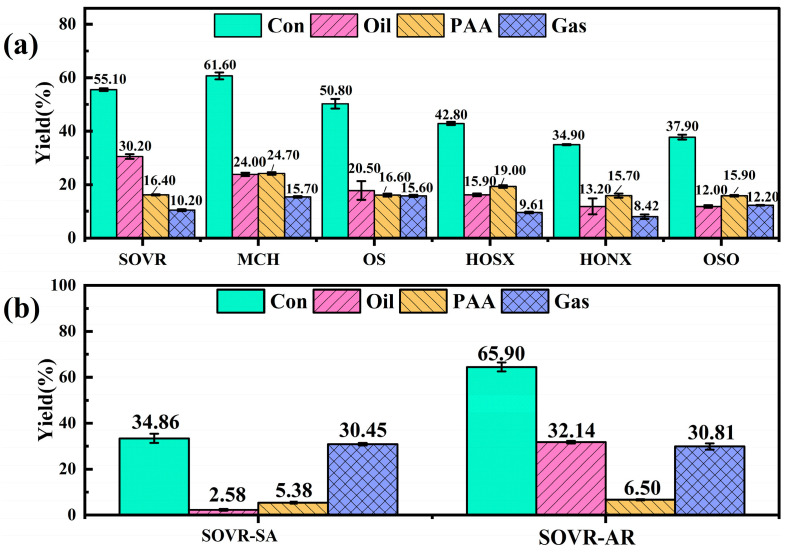
(**a**) Distribution of co-liquefaction yield of heavy oil and (**b**) co-liquefaction yield of heavy oil group components and NMHC.

A co-liquefaction experiment was repeated three times, and the error of each group of experimental data was ≤5%, which is considered indicative of good reproducibility. The yield of the co-liquefaction of the six heavy oils with NMHC is shown in Figure 2a. Taking the oil yield as the evaluation standard, the co-liquefaction reaction performance of the six heavy oils and NMHC was on the order of SOVR (30.20%) > MCH (24.00%) > SO (20.50%) > HONX (15.90%) > HOSX (13.20%) > OSO (12.00%). The oil yield of and group composition content in the six heavy oils were analyzed. It was found that the heavy oils with a high AR content produced a higher co-liquefaction oil yield, which is consistent with the study in [14]. Therefore, the group components of the heavy oil were used as solvents to carry out liquefaction experiments with NMHC for verification.

Among the six heavy oils, SOVR had the best liquefaction performance in COCL. Therefore, the group components of SOVR were selected as solvents to explore its influence on COCL. Because RE and AS have higher viscosities, it is easier to use SA and AR as solvents in coal liquefaction reactors. Therefore, the SA and AR of the SOVR (SOVR-SA and SOVR-AR) were selected as co-liquefaction solvents for NMHC. The distribution of the liquefaction yield is shown in Figure 2b. The data show that, when SOVR-SA was used as the liquefaction solvent, the oil yield was only 2.58%. The yield of SOVR-AR was 32.14%. The results show that the liquefaction performance of SOVR-AR is better than that of SOVR-SA, thus indicating that the AR in heavy oil can promote the COCL process.

### 2.2. Characteristic and Structural Analyses of Heavy Oil

#### 2.2.1. FT-IR Analysis of Heavy Oil

**Figure 3:**
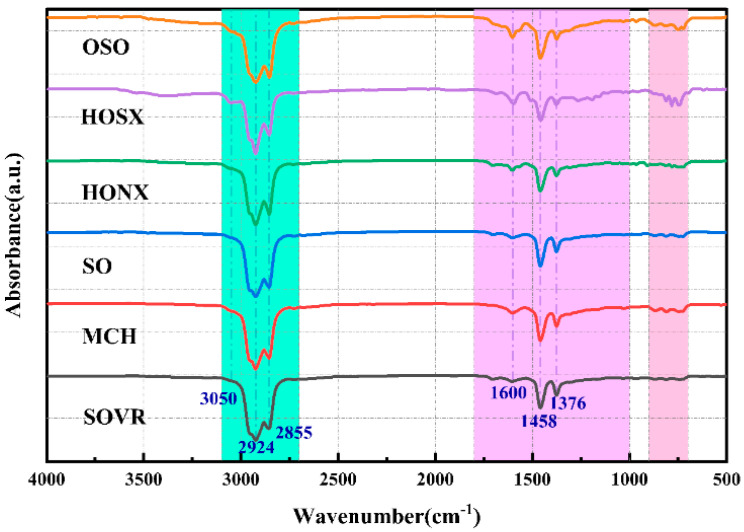
FT-IR spectra of heavy oil.

Fourier transform infrared spectroscopy (FT-IR) is one of the most common techniques used to study the structure and properties of matter [19]. It can provide important information about the chemical structure of materials, especially about the surface functional groups [20]. Figure 3 shows infrared absorption spectra of the six heavy oils. As shown in the figure, the infrared absorption peaks of the six heavy oils mainly appear in the regions of 3100–2800 cm^−1^, 1800–1000 cm^−1^, and 900–700 cm^−1^. The range of 3100–2800 cm^−1^ corresponds to the stretching vibration peaks of aliphatic -CH_2_ and -CH_3_ [21], and the six heavy oils show a strong peak intensity in this range, indicating that they are mainly composed of aliphatic compounds. The characteristic absorption peak corresponding to aromatic ring -C=C- is at 1800–1000 cm^−1^. The absorption peak intensity of the six heavy oils in this region is moderate, which indicates that the aromatic compound content in these six heavy oils is not high. The intensity of the characteristic peak at 1600 cm^−1^ is weak, indicating that the aromatic compounds of the six heavy oils are less condensed [22]. The stretching vibration peak of 900–700 cm^−1^ is a characteristic absorption peak of aromatic substituted compounds. The absorption peaks of the six heavy oils in this region are relatively weak, indicating that there are small numbers of aromatic substituted compounds in the six heavy oils.

In order to quantitatively analyze the functional group contents in the six heavy oils, the infrared spectra of the six heavy oils were fitted in the areas of 3100–2800 cm^−1^, 1800–1000 cm^−1^, and 900–700 cm^−1^ using Origin 2021 software. The fitting results are shown in Table 1. The data show that the aliphatic -CH_3_ contents in OSO, SOVR, and SO were higher, especially in OSO. The content in HONX was lowest. SOVR contained more aliphatic -CH_2_. The aromatic C=C content in the six heavy oils was low: that in HOSX was highest, at 7.02%, and that in OSO was lowest, at 1.22%. The aromatic substituted compound content in the six heavy oils was low. Therefore, the structures of the six heavy oils were mainly composed of aliphatic compounds and they contained small numbers of aromatic compounds and aromatic ring substituted compounds.

#### 2.2.2. ^13^C-NMR Analysis of Heavy Oil

**Figure 4:**
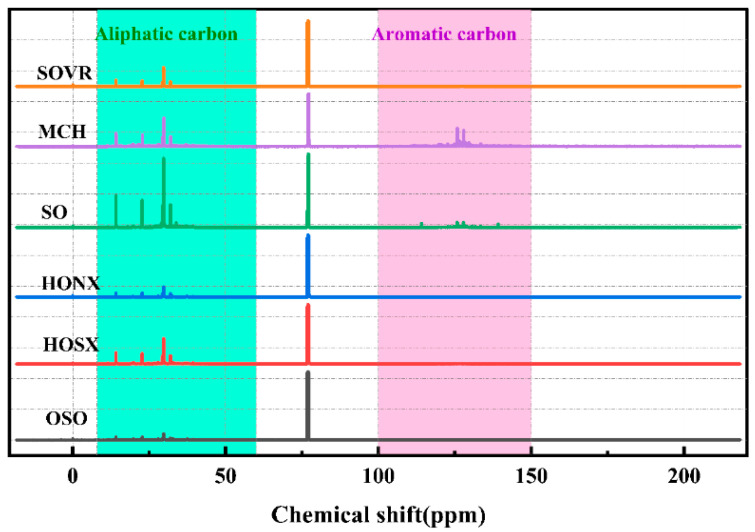
^13^C-NMR spectra of heavy oil.

^13^C-NMR characterization can accurately determine the content of different types of carbon atoms in heavy oil [23]. ^13^C-NMR spectra of the six heavy oils are shown in Figure 4. In order to accurately classify the chemical shifts of the six heavy oils, the “cut-off method” [24] was used to classify their ^13^C-NMR spectra. Then, MestReNova software Mnova 16 was used for area integration to calculate the content of the different types of carbon atoms, and the integration results are shown in Table 2. The analysis results show that the carbon atoms in the six heavy oils can be mainly divided into aliphatic carbon atoms (δ_C_ = 8.0–60.0 ppm) and aromatic carbon atoms (δ_C_ = 100.0–150.0 ppm). The aliphatic carbon atoms can be further divided into three categories: terminal methyl or methyl carbon (f_C1_, 8.0–15.0 ppm) at the γ position and beyond, methyl carbon (f_C2_, 15.0–22.5 ppm) at the α or β position on the aromatic ring, and methylene carbon (f_C3_, 15.0–22.5 ppm) on long-chain fatty acids or cycloalkanes.

Table 2 shows that the f_ali_^C^ contents in the six heavy oils are relatively high, at 44.25%, 50.77%, 53.77%, 56.77%, 59.77%, and 62.77%. The f_ar_^C^ contents in the six heavy oils are 55.75%, 49.24%, 33.25%, 44.32%, 38.46%, and 45.50%, respectively, which are lower than the f_ali_^C^ contents. The results show that the six heavy oils have strong aliphatic structural characteristics, and the basic skeleton is composed of aliphatic carbon. f_C1_ and f_C2_ represent the carbon atoms at the γ position and the α or β position substituents of the aromatic ring, respectively. The sum of f_C1_ + f_C2_ represents the content of aliphatic carbon atoms substituted by aromatic rings. The f_C1_ + f_C2_ contents in the six heavy oils are relatively low, at 7.50%, 9.50%, 11.50%, 13.50%, 15.50%, and 17.50%, indicating that they contain a small number of aromatic ring substituents. This shows that the aliphatic carbon atom content in the six heavy oils is high and that their basic structure is mainly composed of aliphatic carbon skeletons, while there are a small number of aromatic compounds and aromatic ring substituted compounds. This is consistent with the FT-IR analysis results of the six heavy oils.

#### 2.2.3. ^1^H-NMR Analysis of Heavy Oil

**Figure 5:**
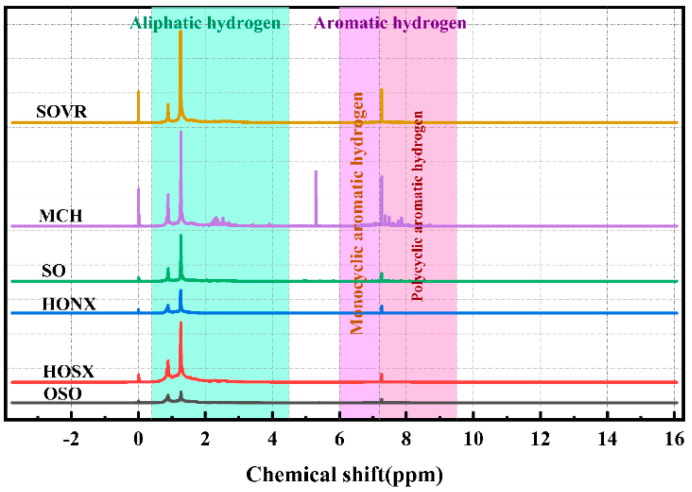
^1^H-NMR spectra of heavy oil.

^1^H-NMR can effectively detect the hydrogen atoms in different chemical environments [25]. Figure 5 shows ^1^H-NMR spectra of the six heavy oils. The hydrogen atoms in the six heavy oils can be mainly divided into hydrogen atoms in aliphatic side chains (δ_H_ = 0.4–4.5 ppm) and hydrogen atoms in aromatic rings (δ_H_ = 6.0–9.5 ppm). The hydrogen atoms in the aliphatic and side chains can be further divided into hydrogen at the γ position of the aromatic ring, hydrogen in the CH_3_ group far away from the γ position, and hydrogen in naphthene CH_3_ (H_γ_, 0.4–1.0 ppm). In addition, there is hydrogen in the beta position of the aromatic ring and hydrogen in the CH_3_, CH_2_, and CH groups far away from the beta position, as well as naphthenic hydrogen (H_β_, 1.0–1.9 ppm) and hydrogen in aromatic α-CH_2_ and α-CH_3_ groups (H_α_, 1.9–4.5 ppm). Aromatic hydrogen includes hydrogen from monocyclic aromatic hydrocarbons (H_A1_, 6.0–7.2 ppm), bicyclic aromatic hydrocarbons (H_A2_, 7.2–7.7 ppm), and tricyclic and higher aromatic hydrocarbon systems (H_A3_, 7.7–9.5 ppm).

^1^H-NMR spectra of the six heavy oils were integrated using MestReNova software, and the integration results are shown in Table 3. The results show that the aliphatic hydrogen atom content in the six heavy oils was high, among which the proportion of H_β_ was highest, with contents of 56.91%, 41.56%, 59.41%, 56.38%, 53.19%, and 54.61%, and SOVR had the highest content. The proportion of H_γ_ in the six heavy oils was relatively low, with contents of 11.68%, 14.58%, 17.80%, 27.13%, 22.80%, and 31.33%, and OSO had the highest content. The H_α_ contents in the six heavy oils were 18.50%, 22.46%, 14.75%, 10.34%, 16.16%, and 9.58%, indicating that all six heavy oils contained a small number of aromatic ring α substituents, among which HOSX had the highest content. The aromatic hydrogen H_A_ contents in the six heavy oils were relatively low, at 12.91%, 21.13%, 8.03%, 6.15%, 7.75%, and 4.47%. Using the modified Brown–Landner formula [26] in an elemental analysis and the GPC data of the six heavy oils, the structural parameters of the six heavy oils were calculated, as shown in Table S2. The molecular weight data of the six heavy oils measured using GPC are shown in Figure S2 and Table S1. The structural parameters showed that the six heavy oils had low aromaticity and a high fatty carbon content and that they existed in the form of naphthenic carbon and alkyl carbon. The low substitution rate of the aromatic rings in these six heavy oils indicated that they contained fewer aromatic substituted compounds. These heavy oils showed strong aliphatic structural characteristics, existed in the form of naphthenes and alkyl carbons, and contained small numbers of aromatic rings and aromatic substituted compounds. This is consistent with the results of the FT-IR and ^13^C-NMR analyses of the six heavy oils.

### 2.3. Evaluation and Analysis of Heavy Oil Co-Liquefaction Performance

From the liquefaction yield data of the six heavy oils and NMHC, it was found that the liquefaction yield differed when different heavy oils were used as solvents. As the selection of a suitable heavy oil for a COCL solvent is helpful in improving crude oil yield, it is very important to evaluate the solvent performance of heavy oil. Referring to the evaluation method of recycle solvent, the PDQIs and δ_C_ of the heavy oils were calculated using NMR data.

**Figure 6:**
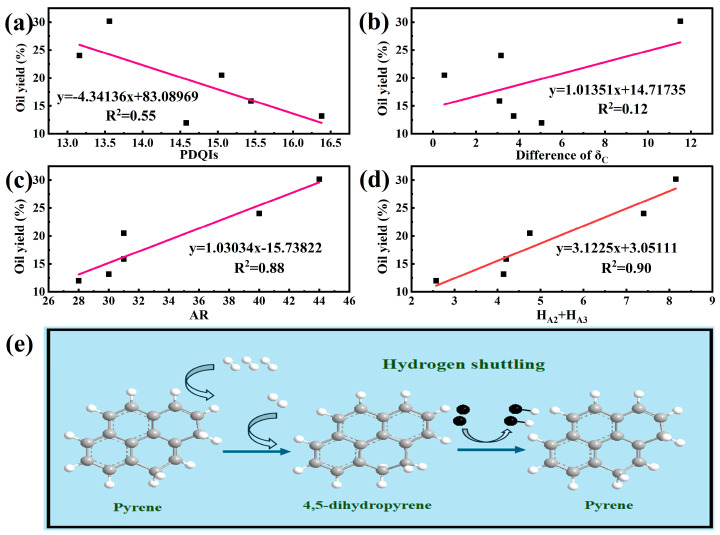
(**a**) PDQIs linear fit to oil yield; (**b**) difference in δ_C_ linear fit to oil yield; (**c**) aromatic component content linear fit to oil yield; (**d**) H_A2_ + H_A3_ linear fit to oil yield; (**e**) hydrogen transfer mechanism of polycyclic aromatic hydrocarbons.

The PDQIs is an evaluation method for recycle solvent, and it is based on the hydrogen supply function of the β-position hydrogen of the cycloalkane ring in hydrogenated aromatic hydrocarbons. It is generally believed that oil yield is positively correlated with the PDQIs value of the solvent [11]. A correlation analysis between the PDQIs and the oil yield of the six heavy oils is shown in Figure 6a. The solubility of coal in solvent indicates the degree to which the coal and solvent are in a favorable reaction state during the interaction. The better the solubility of the solvent to the coal, the better the contact between them and the more complete the reaction [7]. According to the principle of similar compatibility, the closer the δ_C_ of the coal and recycle solvent, the better the mutual solubility and the better the oil yield. A correlation analysis between the δ_C_ and oil yield of the six heavy oils is shown in Figure 6b. Here, the difference between the δ_C_ of the heavy oil and NMHC is fitted with the oil yield. The smaller the difference, the closer the δ_C_ value. If δ_C_ is suitable for evaluating the solvent performance of the heavy oil, then the difference in δ_C_ should be negatively correlated with the oil yield. The PDQIs and δ_C_ data of the six heavy oils are shown in Table S3. The AR in heavy oil has a certain hydrogen supply potential because it contains hydrogenated aromatic hydrocarbons. Heavy oil with a high AR content has better reactivity in COCL and can obtain a higher oil yield. A correlation analysis between the AR and oil yield of the six heavy oils is shown in Figure 6c. Polycyclic aromatic hydrocarbons have a hydrogen transfer ability in the COCL process [27]. Polycyclic aromatic hydrocarbons are hydrogenated by H_2_ to generate hydrogenated aromatic hydrocarbons in COCL, which can effectively provide active hydrogen for the reaction system, and then the hydrogenated aromatic hydrocarbons are converted into polycyclic aromatic hydrocarbons. The mutual transformation of polycyclic aromatic hydrocarbons and hydrogenated aromatic hydrocarbons in COCL promotes the continuous transfer of active hydrogen in the reaction system and improves the yield [28]. Therefore, considering the hydrogenated aromatic hydrocarbons and polycyclic aromatic hydrocarbons together, the hydrogen on these two compounds in heavy oil is represented by H_A2_ + H_A3_ and calculated using ^1^H NMR data. A correlation analysis between H_A2_ + H_A3_ and the oil yield of the six heavy oils is shown in Figure 6d, and its mechanism is shown in Figure 6e.

The data show that the oil production rate of the six heavy oils is neither positively correlated with the PDQIs nor negatively correlated with the δ_C_ difference between the coal and heavy oil. The PDQIs only considers the hydrogen donor property of the hydrogenated aromatic hydrocarbons in recycle solvent and ignores the other components. However, heavy oil contains many components besides hydrogenated aromatic hydrocarbons. δ_C_ only considers the solubility of solvent in coal, ignoring the chemical interaction between the solvent and the coal. Therefore, δ_C_ and the PDQIs may not be suitable for evaluating the solvent performance of heavy oil in COCL. However, the AR and H_A2_ + H_A3_ contents in the heavy oil are positively correlated with the oil yield. There is a strong linear relationship between H_A2_ + H_A3_ and oil yield (R^2^ = 0.90). This shows that the AR content and H_A2_ + H_A3_ are more reliable indices for evaluating the performance of heavy oil solvent in the COCL process.

## 3. Discussion

The structures of the six heavy oils were characterized using FTIR, ^13^C-NMR, and ^1^H-NMR. The data showed that the structures of the six heavy oils were similar, mainly composed of aliphatic compounds and containing a small number of aromatic structures. The recycle solvent used in the coal liquefaction process is usually composed of 2–4 ring hydrogenated aromatic hydrocarbons, which have good hydrogen transfer performance. Therefore, the liquefaction performance of heavy oil as a solvent in COCL is not ideal. δ_C_ only considers the mutual solubility of the solvent and coal and not the reactivity. The PDQIs only considers that the hydrogenated aromatic hydrocarbons contained in the solvent have a good hydrogen supply ability and ignores all components other than hydrogenated aromatic hydrocarbons. Unlike recycle solvent, heavy oil has a complex structure and contains many other components, except for hydrogenated aromatic hydrocarbons. Therefore, the conventional methods for evaluating recycle solvent are not suitable for evaluating the solvent performance of heavy oil in COCL. The AR content in heavy oil was directly proportional to co-liquefaction performance, which is consistent with Flynn’s research results. In this study, this was verified by linearly fitting the AR and liquefied oil yield. In addition, based on the theory that polycyclic aromatic hydrocarbons have a good hydrogen transfer ability in the process of coal liquefaction, the polycyclic aromatic hydrocarbon content (H_A2_ + H_A3_) in the heavy oils was calculated by combining the ^1^H-NMR data of the heavy oils, and it was found that H_A2_ + H_A3_ was directly proportional to the liquefaction performance, with a strong linear relationship (R^2^ = 0.90). This method is more suitable than δ_C_ and the PDQIs for evaluating the solvent performance of heavy oil, and it is simple and effective. Therefore, H_A2_ + H_A3_ can be used as a reliable index to evaluate solvent performance.

## 4. Materials and Methods

### 4.1. Material

The coal sample used in the COCL experiment was NMHC, which is produced in the Naomaohu mining area, Hami, Xinjiang, and is an oil-rich coal suitable for liquefaction. The coal sample was crushed, ground, sieved to 200 mesh, and stored until later use. Industrial and elemental analyses of the NMHC are shown in Table 4.

The solvents used in the COCL experiment consisted of six heavy oils from Xinjiang. SOVR was provided by Xinjiang Changji Chaoyuan Chemical Co., Ltd. (Changji, China), and MCH, SO, HONX, HOSX, and OSO were provided by Xinjiang Turpan Meihuite Petrochemical Products Co., Ltd. (Turpan, China). SO is a typical unconventional oil product, which is exploited by shale resources in Jimsar, Xinjiang. SOVR is a typical heavy oil distillate obtained from shale oil using vacuum distillation technology. MCH is obtained via the dry distillation of coal and is a coal tar. HONX is an oil product produced in the Junggar Basin in northern Xinjiang, where it is a typical heavy crude oil. HOSX is an oil product produced in the Tarim Basin in southern Xinjiang, where it is a typical heavy crude oil. OSO is a typical heavy oil extracted from oil sands resources in the Junggar and Tarim Basins, Xinjiang. The elemental analysis results of the six heavy oils are shown in Table 5. Fe_2_O_3_, S, tetrahydronaphthalene, n-hexane, tetrahydrofuran, n-heptane, toluene, and dichloromethane were provided by Tianjin Zhiyuan Chemical Reagent Co., Ltd. (Tianjin, China).

### 4.2. Experimental Process and Analytical Method

#### 4.2.1. COCL Experiment

COCL experiments were performed using a 100 mL autoclave. The coal sample used in the experiment was NMHC. In order to avoid the influence of different experimental conditions, the reaction conditions of the DCL experiment of the same coal sample were set according to the literature [29]. Dry ash-free coal (7 g), Fe_2_O_3_ catalyst (0.3 g), S co-catalyst (0.24 g), and solvent (14 g) were added to an autoclave in turn. Here, the solvents included six types of heavy oil, as well as the SA and AR components in the SOVR. The initial hydrogen pressure was 7 MPa, the reaction temperature was 430 °C, the stirring speed was 400 r/min, and the reaction time was 1 h. After the reaction, the autoclave was cooled with a blower, and the temperature dropped below 300 °C within 5 min. Solid–liquid products were continuously extracted and separated via Soxhlet extraction with n-hexane and tetrahydrofuran for 36 h and 8 h, respectively. The n-hexane-soluble substance was liquefied oil, the n-hexane-insoluble and tetrahydrofuran-soluble substances were asphaltenes, and the tetrahydrofuran-insoluble substance was the residue. Please refer to GB/T33690-2017 [30] for the calculation methods of the raw material conversion rate, oil yield, gas yield, asphaltene yield, and hydrogen consumption. The calculation formulas are as follows:

η_oil_ = Conv. + DHC − η_Res._ − η_AP_ − η_Gas_ (1)

(2) $$\begin{array}{c} \eta_{\mathrm{Gas}}=\frac{\left(P_{0}+P_{2}\right)\times \left(V_{0}-V_{1}\right)\times T_{0}}{P_{0}\times T_{2}\times V_{m}\times M_{\mathrm{RM}}}\times \sum_{i}\left(\frac{R_{i}U_{i}}{100}\right)\;\times \;100\%\; \end{array}$$

(3) $$\begin{array}{c} \eta_{AP}=\frac{M_{\mathrm{AP}}}{M_{\mathrm{RM}}}\;\times \;100\% \end{array}$$

(4) $$\begin{array}{c} \eta_{\mathrm{Res}.}=\frac{M_{\mathrm{THFIS}}-M_{\mathrm{Cat}.}-M_{\mathrm{As}h}}{M_{\mathrm{RM}}} \end{array}$$

(5) $$\begin{array}{c} DHC=\left(\frac{P_{0}+P_{1}}{T_{1}}-\frac{P_{0}+P_{2}}{T_{2}}\times \frac{R_{H_{2}}}{100}\right)\times \frac{T_{0}\times M_{H_{2}}\times \left(V_{0}-V_{1}\right)}{P_{0}\times V_{m}\times M_{\mathrm{RM}}} \end{array}$$

(6) $$\mathrm{Conv}.=100\;-\;\eta_{\mathrm{Res}.}$$

In formulas (1)–(6), η_oil_, η_Gas_, η_AP_, η_Res._, DHC, and Conv. are the oil yield, gas yield, asphaltene yield, residue rate, H_2_ consumption, and conversion rate, respectively. M_THFIS_, M_Cat._, M_Ash_, and M_AP_ are the tetrahydrofuran-insoluble substance, the sum of the mass of the catalyst and co-catalyst, and the mass of the ash and asphaltene (g), respectively; P_0_, P_1_, and P_2_ are the local atmospheric pressure, initial hydrogen pressure, and pressure in the kettle after cooling after the reaction (MPa), respectively; T_0_ is 273.15 k; T_1_ and T_2_ are the initial temperature of the reaction and the temperature (k) after cooling after the reaction, respectively; V_0_ and V_1_ are the effective and material volumes (L) of the autoclave, respectively; Vm is 22.4 L/mol; R_i_ is the volume fraction of gas i (excluding H_2_); U_i_ is the relative molecular mass of i gas (g/mol); and M_RM_ is the dry and ash-free basis mass of the NMHC mass (g).

#### 4.2.2. Characterization of Heavy Oil

The functional group structures of the heavy oils were analyzed using a Bruker VERTEX 70 RAMI Fourier transform infrared spectrometer (FT-IR) from Germany. The test conditions were as follows: a scanning band of 4000–400 cm^−1^, a wave number accuracy of 4 cm^−1^, and a cumulative scanning time of 16 times.

Different types of carbon and hydrogen atoms in the heavy oil were analyzed using a Bruker 600 MHz superconducting nuclear magnetic resonance spectrometer (NMR) from Germany.

The ^1^H-NMR test conditions were as follows: room temperature, deuterated chloroform (CDCl_3_) as the solvent, tetramethylsilane (TMS) as the internal standard, a resonance frequency of 399.740 MHz, a scanning width of 6 kHz, a sampling rate of 20 times, a sampling time of 3.744 s, and a delay time of 10 s.

The ^13^C-NMR test conditions were as follows: room temperature, deuterated chloroform (CDCl_3_) as the solvent, tetramethylsilane (TMS) as the internal standard, a resonance frequency of 100.525 MHz, a scanning width of 25 kHz, a sampling rate of 21,500, a sampling time of 1.199 s, and a delay time of 5 s.

## 5. Conclusions

In summary, the structures of six heavy oils were mainly composed of aliphatic compounds, with small numbers of aromatic and aromatic substituted compounds. Among them, SOVR showed the highest oil yield in the COCL process. In the COCL experiment, the liquefaction performance of SOVR-AR was better than that of SOVR-SA, which shows that AR in heavy oil plays a key role in improving COCL efficiency. When evaluating the solvent performance of the COCL heavy oil, it was found that the PDQIs and δ_C_ were not effective indicators. In contrast, AR and H_A2_ + H_A3_ had a good correlation with oil yield. In particular, the correlation between H_A2_ + H_A3_ and oil yield was stronger, and the R^2^ value was 0.90. In this study, it was confirmed that the AR content and H_A2_ + H_A3_ value of heavy oil could be used as methods to evaluate the solvent performance in COCL, thus providing a theoretical reference for selecting a suitable heavy oil and improving liquefaction reaction efficiency when using COCL technology.

## Figures and Tables

**Table 1:** FTIR attribution and quantitative analysis of heavy oil.

Band Position/cm^−1^	Functional Group	Area Percentage/%					
		SOVR	MCH	SO	HONX	HOSX	OSO
2950–2930	Aliphatic -CH_3_	57.61	54.64	57.90	55.15	37.44	60.84
2870–2850	Symmetric aliphatic -CH_2_	22.05	14.30	19.66	13.38	14.36	17.53
1600	Aromatic C=C	2.33	4.32	2.76	2.75	7.02	1.22
1480–1400	Asymmetric CH_3_, CH_2_	9.66	13.19	11.27	9.28	9.50	11.93
1400–1240	Symmetric deformation -CH_3_	1.95	4.79	5.20	16.29	25.54	5.62
900–860	Five adjacent H deformations	0.55	0.00	0.27	0.69	1.07	0.67
860–810	Four adjacent H deformations	0.76	1.80	0.58	0.79	1.95	0.65
810–750	Three adjacent H deformations	3.86	2.36	0.47	0.00	2.37	0.91
750–720	Two adjacent H deformations	1.23	4.60	1.89	1.66	0.74	0.63

**Table 2:** Integral attribution and content of ^13^C-NMR spectra of heavy oil.

Symbol	δ_C_	Relative Content (%)					
		SOVR	MCH	SO	HONX	HOSX	OSO
f_ali_^C^	8.0–60.0	44.25	50.77	53.77	56.77	59.77	62.77
f_C1_	8.0–15.0	3.57	4.57	5.57	6.57	7.57	8.57
f_C2_	15.0–22.5	3.93	4.93	5.93	6.93	7.93	8.93
f_C3_	22.5–60.0	36.75	37.75	38.75	39.75	40.75	41.75
f_ar_^C^	100.0–150.0	55.75	49.24	33.25	44.32	38.46	45.50
f_C1_ + f_C2_	-	7.50	9.50	11.50	13.50	15.50	17.50

**Table 3:** Integral attribution and content of ^1^H-NMR spectra of heavy oil.

Symbol	δ_H_	Relative Content (%)					
		SOVR	MCH	SO	HONX	HOSX	OSO
H_γ_	0.4–1.0	10.99	17.94	17.80	26.51	23.54	31.33
H_β_	1.0–1.9	57.49	44.03	59.41	57.33	53.49	54.61
H_α_	1.9–4.5	18.99	24.80	14.75	9.996	16.24	9.58
H_A_	6.0–9.5	12.54	13.23	8.03	6.20	6.73	4.47
H_A1_	6.0–7.2	4.39	5.83	3.28	2.00	2.59	1.90
H_A2_	7.2–7.7	2.31	3.16	2.09	1.45	1.01	1.07
H_A3_	7.7–9.5	5.84	4.24	2.66	2.75	3.13	1.50
H_A2_ + H_A3_	-	8.15	7.4	4.75	4.2	4.14	2.57

**Table 4:** Industrial and elemental analyses of NMHC.

Industrial Analysis w/%				Elemental Analysis wdaf/%					H/C (mol Ratio)
Mad	Ad	Vdaf	FCdaf	C	H	N	S	Oa	
10.58	6.51	48.97	42.66	73.76	6.16	0.82	0.48	18.78	1.00

Difference reduction.

**Table 5:** Elemental analyses of heavy oil.

Sample	Elemental Analysis wdaf/%					H/C (mol Ratio)
	C	H	O	N	S	
SOVR	85.87	12.09	<0.5	2.07	<0.5	1.69
MCH	85.12	13.68	<0.5	1.25	<0.5	1.93
SO	85.05	13.68	<0.5	1.25	<0.5	1.93
HONX	85.36	13.48	<0.5	0.68	0.99	1.89
HOSX	85.16	12.85	<0.5	0.43	2.29	1.81
OSO	86.33	13.08	<0.5	1.08	<0.5	1.82

## Data Availability

Data will be made available on request.

## Supplementary Materials

The following supporting information can be downloaded at https://www.mdpi.com/article/10.3390/ijms26136048/s1.
