# Supplementary material for: Solvent Performance Evaluation of Heavy Oil in Coal–Oil Co-Liquefaction
Source: Int J Mol Sci. 2025 Jun 24;26(13):6048. doi: 10.3390/ijms26136048 (PMC12250110; doi:10.3390/ijms26136048)
Supplement: Supplementary file 1 [file ijms-26-06048-s001.zip › ijms-3553938-supplementary.pdf]

Supplementary Materials

1. Separation process of heavy oil group composition

Six kinds of heavy oils were separated by column chromatography. The extraction solvent was n-heptane and toluene, and the elution solvent was n-heptane (80 mL), and the mixture of toluene (80 mL) and toluene and ethanol (40 mL each). The mass of each oil sample was 1 g. The separation process is shown in Figure S1.

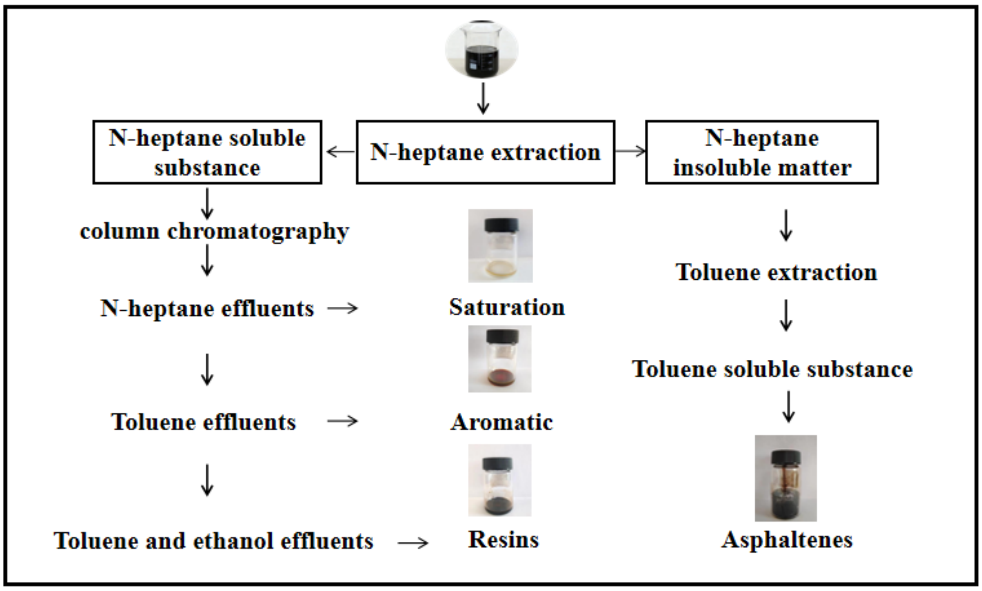

Figure S1. Group composition separation process.

2. GPC analysis of six heavy oils

Gel permeation chromatography (GPC) can be used to obtain the number average molecular weight (MN) and weight average molecular weight (MW) of these six heavy oils. Fig. S2 shows the GPC spectra of six heavy oils, and Table S1 shows the GPC results of six heavy oils. Because of the complex structural composition of heavy oil, it is more accurate to express the relative molecular weight by weight average molecular weight (MW).

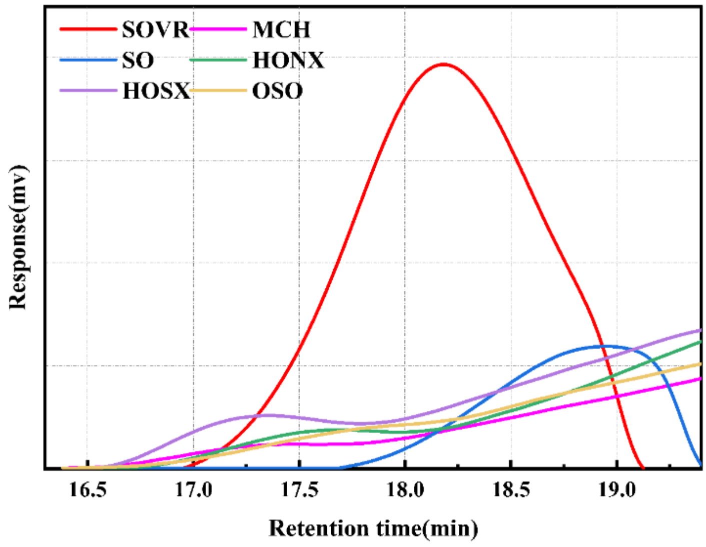

Figure S1. GPC spectra of heavy oil.

From the GPC spectrum and PDI results of six heavy oils, the distribution of SO and SOVR is

concentrated, and there is little structural difference. In contrast, MCH, HONX, HOSX and OSO show higher PDI values, indicating that these four oil samples have wider molecular distribution and greater structural variability.

Table S1 GPC Consequence of heavy oil

Sample	Mn(g·mol^{-1})	Mw(g·mol^{-1})	PDI(Mw/Mn)
SOVR	528	674	1.28
MCH	584	1715	2.94
SO	355	423	1.19
HONX	673	2049	3.04
HOSX	650	2089	3.21
OSO	651	2263	3.48

3. Structural parameters of heavy oil

According to elemental analysis, gel permeation chromatography and 1H NMR data, the structural parameters of six heavy oils were calculated by using the modified Brown-Landner formula, as shown in Table S2.

Table S2 Heavy Oil Structural Parameters.

symbol	description	value					
		SOVR	MCH	SO	HONX	HOSX	OSO
f_A	aromaticity	0.27	0.19	0.12	0.12	0.17	0.14
σ	hydrogen substitution rate around aromatic rings	0.42	0.35	0.48	0.46	0.51	0.52
M	Average molecular weight	674	1588	423	2049	2089	2263
C_T	Total carbon number	48.19	112.55	29.96	145.63	148.13	162.67
H_T	total hydrogen number	80.85	215.54	57.41	274.04	266.33	293.68
C_S	Alkyl carbon number	35.21	91.48	26.40	128.59	122.71	140.26
C_N	naphthenic carbon number	16.57	24.47	21.70	31.56	28.71	36.58
C_P	linear alkyl carbon number	18.63	67.00	4.69	97.03	94.01	103.68
C_A	aromatic carbon number	12.98	21.07	3.56	17.04	25.41	22.41
R_T	Total ring number	6.89	10.89	5.81	11.65	13.03	14.25
R_A	aromatic ring number	2.75	4.76	0.39	3.76	5.85	5.10
R_N	number of cycloalkanes	4.14	6.12	5.42	7.89	7.18	9.15
f_N	naphthenic carbon ratio	0.34	0.22	0.72	0.22	0.19	0.22
f_P	linear alkyl carbon ratio	0.37	0.60	0.16	0.67	0.63	0.64
L	average chain length	5.91	5.92	1.38	3.91	4.64	3.38

4. PDQI and δC calculation

According to the elemental analysis data and nuclear magnetic resonance data of six heavy oils, the PDQI and solubility parameter δC are calculated as follows:

$$PDQI = \frac{H_\beta}{H_t} \times H\% \times 10 \tag{6}$$

Where H_β is the integral value of 1.5-2.0 ppm in ^{1}H-NMR, Ht is the integral value of all hydrogen, and H% is the mass fraction of H atom.

$$\delta_C = 2.045 \times \frac{7.0 + 63.5 \times f_a + 63.5(\frac{H}{C}) + 106(\frac{O}{C}) + 51.8(\frac{N+S}{C})}{-10.9 + 12 \times f_a + 13.9(\frac{H}{C}) + 5.5(\frac{O}{C}) - 2.8(\frac{N+S}{C})} \tag{7}$$

Where H/C is the ratio of hydrogen to carbon atoms, O/C is the ratio of oxygen to carbon atoms, and (N+S)/C is the ratio of nitrogen and sulfur atoms to carbon atoms, fa is the aromaticity. The calculated results of PDQI and δC of six heavy oils are shown in Table S3. The δ_C of NMHC is 25.99.

Table S3 Heavy Oil PDQI and δ_C

**Sample**	**PDQI**	**δ_C**
SOVR	13.56	37.50
MCH	13.16	29.15
SO	15.05	26.52
HONX	15.44	29.08
HOSX	16.38	29.74
OSO	14.58	31.04
